# 3D-MRI analysis of cartilage thickness changes after PRP injection in medial knee osteoarthritis: A preliminary report

**DOI:** 10.1371/journal.pone.0321067

**Published:** 2025-04-30

**Authors:** Ichiro Sekiya, Hisako Katano, Mitsuru Mizuno, Kentaro Endo, Asuka Asami, Michiko Kajiwara, Naoki Otomo, Hideyuki Koga, Jun Masumoto, Nobutake Ozeki

**Affiliations:** 1 Center for Stem Cell and Regenerative Medicine, Institute of Science, Tokyo, Japan; 2 Blood Transfusion and Cell Therapy Center, Institute of Science, Tokyo, Japan; 3 Department of Joint Surgery and Sports Medicine, Graduate School of Medical and Dental Sciences, Institute of Science, Tokyo, Japan; 4 Fujifilm Corporation, Tokyo, Japan; University of Naples Federico II: Universita degli Studi di Napoli Federico II, ITALY

## Abstract

The regenerative effect of platelet-rich plasma injection on cartilage in knee osteoarthritis remains controversial. The purpose of this study was to use our recently developed 3D-MRI evaluation system to examine in detail the changes in cartilage thickness occurring six months after platelet-rich plasma injection. This study included 21 knees from 16 patients with medial knee osteoarthritis. An autologous protein solution (APS) was injected as platelet-rich plasma, and magnetic resonance imaging scans were taken before and six months after the injection. Cartilage thickness was quantified in seven regions using SYNAPSE 3D. Based on previous studies, the measurement error was set at 0.1 mm. The proportion of knees in which cartilage thickness increased (>0.1 mm) was highest in the anteromedial femoral region (43%); followed by the anterolateral femoral and lateral tibial regions (24%); the posterolateral femoral, patellar, and medial tibial regions (19%); and lowest in the posteromedial femoral region (14%). Notably, in the posteromedial femoral and medial tibial regions, which are primarily affected by medial osteoarthritis, less than 20% of the knees showed increased cartilage thickness. Our findings suggest that while platelet-rich plasma injection may have a positive effect on cartilage thickness in certain regions of the knee, its impact on the regions most affected by medial osteoarthritis appears limited.

## Introduction

Osteoarthritis of the knee ravages joint cartilage, progressively causing pain and functional impairment [1]. However, the administration of platelet-rich plasma (PRP) therapy has emerged as a treatment for this condition [2,3]. PRP, which is a concentrate of platelets derived from the patient’s own blood, is rich in growth factors that are primarily thought to contribute to inflammation reduction. Therefore, PRP therapy offers hope for symptom improvement in patients with knee osteoarthritis. However, the effects of PRP as a knee osteoarthritis therapy still require evaluation from both structural and clinical perspectives.

While patient-reported outcome measures such as the Knee Injury and Osteoarthritis Outcome Score (KOOS) are widely used to assess clinical improvements in knee osteoarthritis [4], the regenerative effects of PRP on cartilage remains controversial, with no clear consensus. Critical clinical studies evaluating the cartilage structure–improving effects of PRP have mainly included two-dimensional magnetic resonance imaging (2D-MRI) evaluations [5–8], qualitative MRI assessments [9], and three-dimensional MRI (3D-MRI) analysis [10,11]. Crucially, 2D-MRI evaluation and qualitative MRI evaluation fall short in assessing the entire articular cartilage, whereas conventional 3D-MRI evaluation has posed some challenges, such as the manual extraction of cartilage regions and insufficient explanations of the methods used for evaluation.

We have recently revolutionized 3D-MRI analysis by developing an artificial intelligence (AI)-powered system that automatically extracts cartilage from MRI images, creates 3D images, and quantifies the cartilage thickness by region. Leveraging this advanced system, we have conducted several epidemiological studies aimed at unveiling the pathophysiology of knee osteoarthritis [12–16]. The use of this system has enabled us to validate the accuracy and reproducibility of MRI-based cartilage thickness measurements [17,18]. The purpose of the present study was to use this 3D-MRI evaluation system to evaluate the changes in cartilage thickness 6 months after PRP injection and to assess the clinical outcomes using the KOOS values. The study findings were expected to provide a more accurate understanding of the effects of PRP on both cartilage structure and clinical symptoms and to demonstrate more clearly the efficacy and limitations of PRP therapy in the treatment of medial knee osteoarthritis.

## Materials and methods

### Ethics approval and consent to participate

This study was conducted in accordance with the Declaration of Helsinki and was approved by the Bureau of Health and Welfare of the Japanese Ministry of Health, Labour, and Welfare after review by the Certified Special Committee for Regenerative Medicine at our university. Informed consent was obtained from all study subjects. The study included all consecutive patients who received PRP injections for knee osteoarthritis at our institution between May 2021 and April 2024. Data were accessed for research purposes beginning on May 1st, 2024. The authors had access to information that could identify individual participants during and after data collection. All identifiable information was stored in password-protected files on secure servers, accessible only to authorized research personnel. The data was anonymized for analysis and publication purposes.

### Patient selection

The main inclusion criteria for this study were individuals diagnosed with knee osteoarthritis, those who were at least 20 years old at the time of consent, and those in good overall health. Patients were specifically selected if they met the following criteria: no previous PRP treatment, ineffective response to or unwillingness to continue pain medications, failure to respond to at least 3 months of lifestyle guidance and home exercises, no history of surgery on the target knee, and no desire for future surgical intervention. All patients voluntarily opted for this self-funded PRP treatment. The main exclusion criteria were individuals with active inflammation, those with current or past malignant tumors, those with serious comorbidities, and women who were pregnant or possibly pregnant.

### Kellgren–Lawrence (KL) grading

Radiographs were taken of the anteroposterior view of the knee as the patient stood with full knee extension. Medial knee osteoarthritis was graded according to the modified Kellgren–Lawrence (KL) grade described by Guermazi et al. [19]. A KL grade of 0 shows no features of osteoarthritis, KL grade 1 shows osteophytic lipping (equivocal osteophytes), KL grade 2 shows definite (unequivocal) osteophytes, KL grade 3 shows joint space narrowing, and KL grade 4 shows a definite deformity of the bone ends (bone-to-bone appearance) [15].

### PRP preparation

The PRP used was an autologous protein solution (APS). The APS was prepared using the nSTRIDE APS Kit (Zimmer Biomet). In the first step, 55 mL of blood and 4 mL of anticoagulant citrate dextrose solution A (Citra Labs, Braintree, MA) were injected into the nSTRIDE Cell Separator, and approximately 6 mL of PRP was separated after centrifugation at 3200 rpm for 15 min. The prepared PRP was then transferred to the second step involving the nSTRIDE Concentrator, where it was exposed to polyacrylamide beads and filtered by centrifugation at 2000 rpm for 2 min to produce approximately 2–3 mL of APS [20,21]. APS was injected intra-articularly using an 18G or 21G needle within 30 min of preparation. After joint puncture, as much joint fluid as possible was first aspirated, and then the APS was injected. On the day of the injection, the patients were prohibited from taking baths or engaging in strenuous exercise. No specific restrictions were imposed from the following day onward. The use of insoles and/or braces was neither specifically recommended nor prohibited.

### Lifestyle guidance and home exercise program

All patients received standardized osteoarthritis-related lifestyle guidance [22] at least 3 months prior to the injection, but without implementing formal physical therapy. Patients were instructed to perform three specific home exercises: setting, calf pumping, and hip lifting. Follow-up visits were conducted at 1 and 3 months post-injection to monitor progress and ensure compliance with the exercise program. Throughout the 6 month observation period, patients continued their prescribed pain medications and home exercises as needed.

### MRI scanning

MRI was performed at 3.0 T (Achieva 3.0TX, Philips, Amsterdam, Netherlands) with a 16-channel coil. Images in the sagittal plane of the knee joint were acquired with both a fat-suppressed spoiled gradient echo sequence image (SPGR) and a proton density weighted (PDW) image (Table 1) [16,17]. MRI scans were performed before the PRP injection and 6 months after the injection.

**Table 1:** Imaging parameters for MRI sequences.

	SPGR	PDW
Repetition time (msec)	20	1000
Echo time (msec)	1st: 7	35
	2nd: 13.8	
Flip angles (°)	35	90
Echo train length	(−)	32
Acquisition voxel size (mm)	0.6 × 0.6 × 0.6	0.6 × 0.6 × 0.6
Reconstruction matrix size (mm)	0.3 × 0.3 × 0.3	0.3 × 0.3 × 0.3
No. of slices	320	320
Slice thickness (mm)	0.3	0.3
Slice gap (mm)	0	0
Field of view (mm × mm)	150 × 150	150 × 150
WFS/BW (pix/Hz)	2.002/217.0	0.836/519.4
Number of excitations	1	1
Total examination time	7 min 30 sec	7 min 10 sec

SPGR = fat-suppressed spoiled gradient echo; PDW = proton density weighted; WFS/BW = actual waterfat shift/bandwidth; MRI = magnetic resonance imaging.

### Automatic segmentation

MRI analyses using deep-learning-based segmentation were performed using 3D volume analysis software (SYNAPSE 3D [Japanese product name: SYNAPSE VINCENT], version 6.8, Fujifilm Corporation, Tokyo, Japan). The automatic segmentation algorithm was trained on 101 datasets. The bone region was automatically segmented from the PDW images, and the cartilage region was automatically segmented from the SPGR images. The 3D image was then reconstructed [16,17].

### Measurements of cartilage thickness

**Fig 1:**
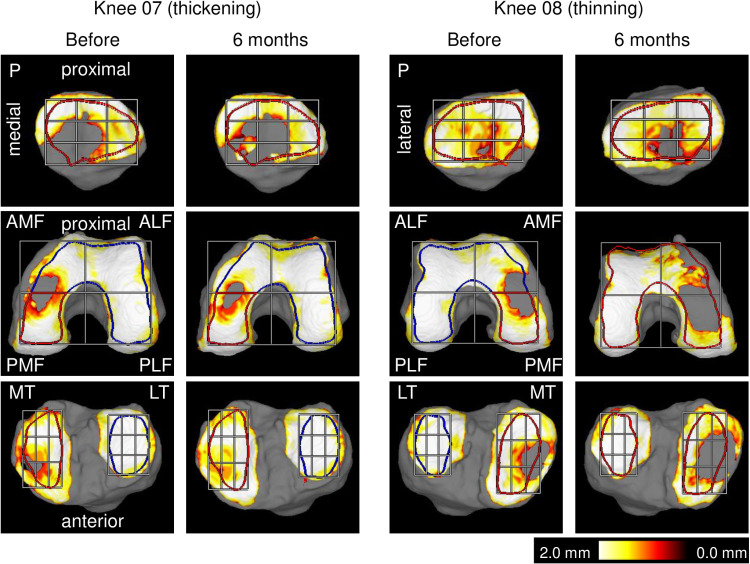
Reconstructed 3D MRI for knee cartilage and ROIs for quantification of cartilage thickness. Knee 07 is a representative case in which the cartilage thickness increased in the PMF and MT regions. Knee 08 is a representative case in which the cartilage thickness decreased in the PMF and MT regions. P, patellar; AMF, anterior medial femoral; ALF, anterior lateral femoral; PMF, posterior medial femoral; PLF, posterior lateral femoral; MT, medial tibial; LT, lateral tibial. Note: The P, MT, and LT regions are divided into 9 sections for reference. The patella is inverted mediolaterally to show its relationship with the femur.

The cartilage region of interest (ROI) was derived by creating 3D images of the bone, followed by definition based on the bone’s shape and the smoothness of its surface. Regions located 2 mm inside this defined cartilage area were set as the ROI for measuring cartilage thickness (Fig 1).

The femoral cartilage was projected along the long axis of the femur. The rotation was determined by generating a horizontal tangent line between the ROI at the posteromedial cartilage and the ROI at the posterolateral cartilage. The software drew lines that split the ROI equally in the longitudinal and transverse directions, resulting in four regions. The tibial cartilage was also projected along the long axis of the tibia, and the ROIs for the medial and lateral tibias were automatically drawn as two closed curve lines [16]. The patellar cartilage was projected so that the area reached its maximum, and the ROI was automatically drawn as a closed curve line [13].

A visual map of the cartilage thickness was provided using a color scale, with thicker areas indicated in white and thinner areas indicated in red. The software segmented the cartilage overlaying the most superficial layer of the bone into the smallest detectable units, quantified the thickness for each unit, and provided the average cartilage thickness of the ROI.

The ROIs of the projected 3D images taken before and 6 months after the injection were compared separately for the femur, tibia, and patella. If one of the ROIs was inappropriate, the ROI from the other image was copied onto it. If both ROIs were inappropriate, one was manually corrected and then copied to the other. The modified ROIs are shown in the results.

### Evaluation of clinical scores

The KOOS values were evaluated before and 6 months after PRP injection [23].

### Statistical analysis

Pearson’s correlation coefficient was used to evaluate the association between changes in cartilage thickness over 6 months and the subscale KOOS changes, patient age, and KL grade. A P-value <0.05 was considered statistically significant; however, the significance level was adjusted using the Bonferroni correction to account for multiple testing. The analysis was performed using the BellCurve software for Excel (Social Survey Research Information Co., Ltd., Tokyo, Japan).

## Results

### Patient enrollment

**Fig 2:**
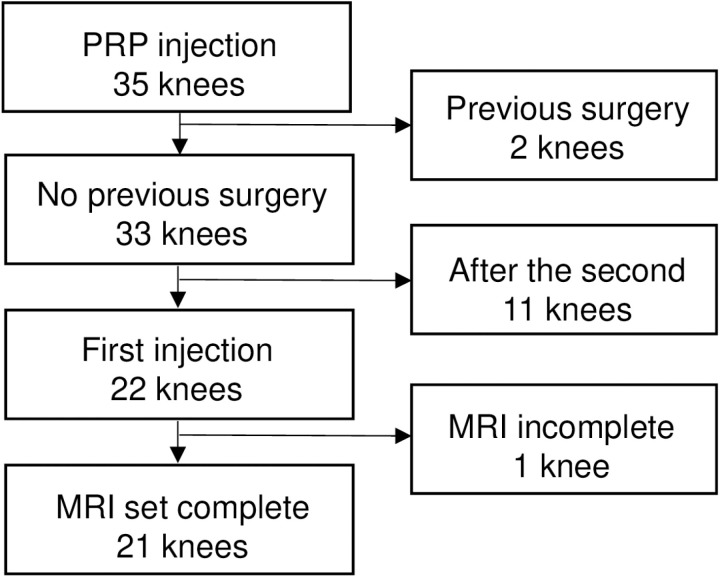
Enrollment of patients. The study included 21 knees that had no history of knee surgery and were receiving PRP injection for the first time.

PRP injections were administered to 35 knees during the relevant period (Fig 2). Two cases with a history of knee surgery (medial meniscectomy and high tibial osteotomy) were excluded. After excluding cases requiring a second or subsequent injections and one knee with an incomplete MRI examination, 21 knees of 15 patients were included in the study. All cases were diagnosed as medial osteoarthritis, with 5 knees classified as KL 1 or 2, and 16 knees as KL 3 or 4 (Table 2).

**Table 2:** Patient population.

Gender	Female	9	subjects
	Male	6	subjects
	Total	15	subjects
Age	Median	63	years
	Maximum	80	years
	Minimum	36	years
BMI	Median	25.4	kg/m^2^
	Maximum	31.7	kg/m^2^
	Minimum	19.3	kg/m^2^
Laterality	Unilateral	9	subjects
	Bilateral	6	subjects
KL	Grade 1	1	knee
	Grade 2	4	knees
	Grade 3	11	knees
	Grade 4	5	knees
	Total	21	knees

The study included a total of 15 patients: 6 patients received injections in both knees simultaneously, while 9 patients received injections in one knee (Table 2). Of these 15 patients, 9 were female and 6 were male, ranging in age from 36 to 80 years, with a median age of 63 years. The patient BMI ranged from 19.3 to 31.7, with a median of 25.4.

### Changes in cartilage thickness 6 months after PRP injection

**Fig 3:**
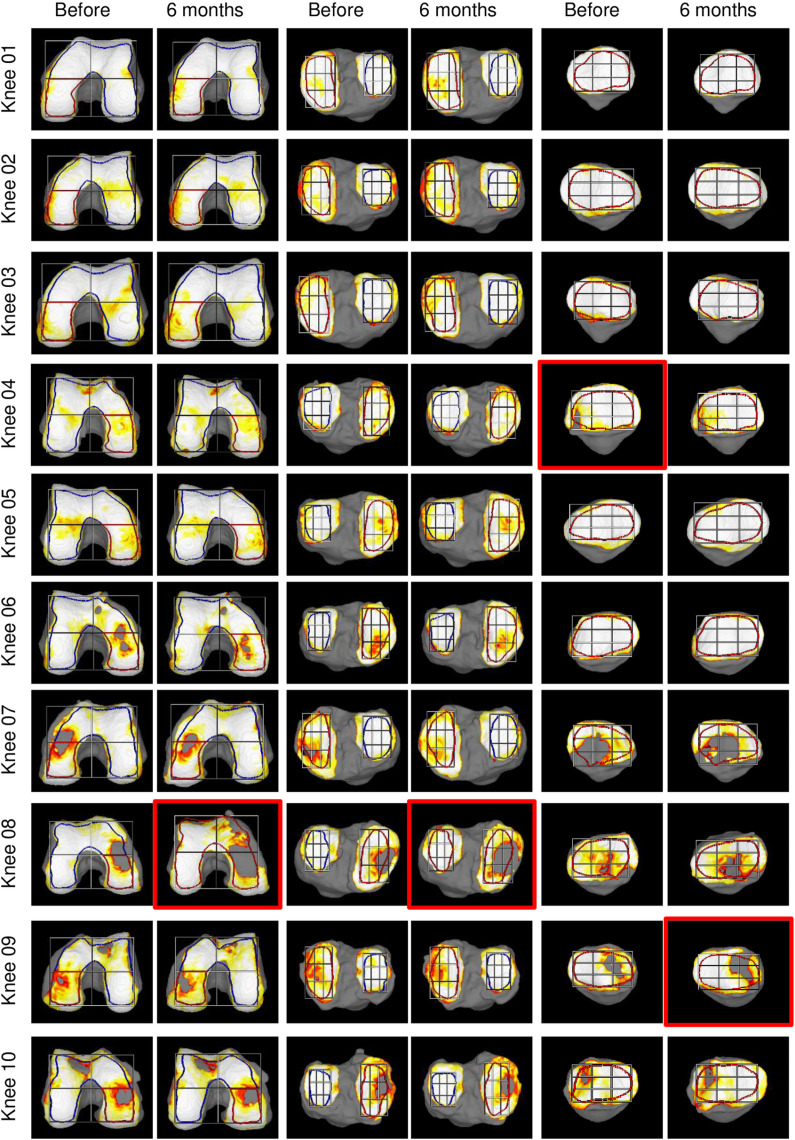
Reconstructed 3D MRI for knee cartilage and ROIs in Knee 01-10, representing the first half of the complete series. The 21 knees are arranged in descending order of cartilage thickness in the PMF region. The red frame indicates cases in which the automatic ROI setting was inappropriate, so another ROI was copied. The blue frame indicates cases in which the automatic ROI settings for two areas were inappropriate, so one was set manually and the other was copied from it.

**Fig 4:**
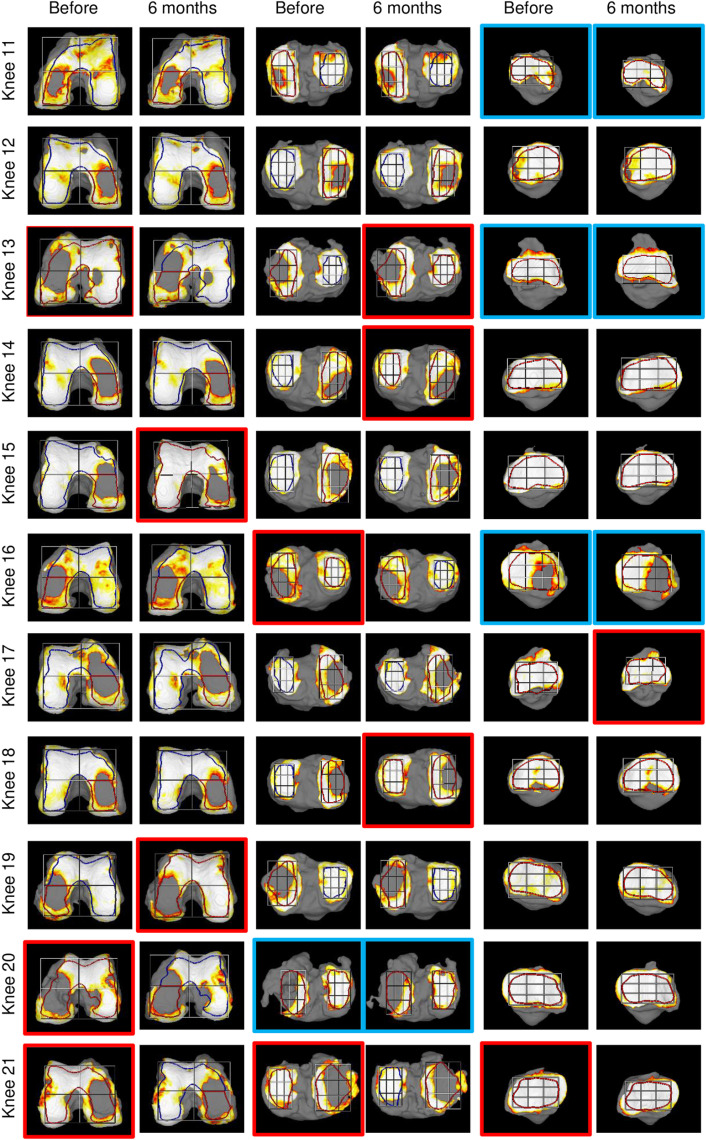
Reconstructed 3D MRI for knee cartilage and ROIs in Knee 11-21, representing the second half of the complete series. All 21 knees (across Fig 3 and Fig 4) are arranged in descending order of PMF cartilage thickness. Red frames indicate inappropriate automatic ROI settings requiring copying, while blue frames show cases where ROI settings for two areas were inappropriate, requiring manual setting of one area and copying to the other.

**Fig 5:**
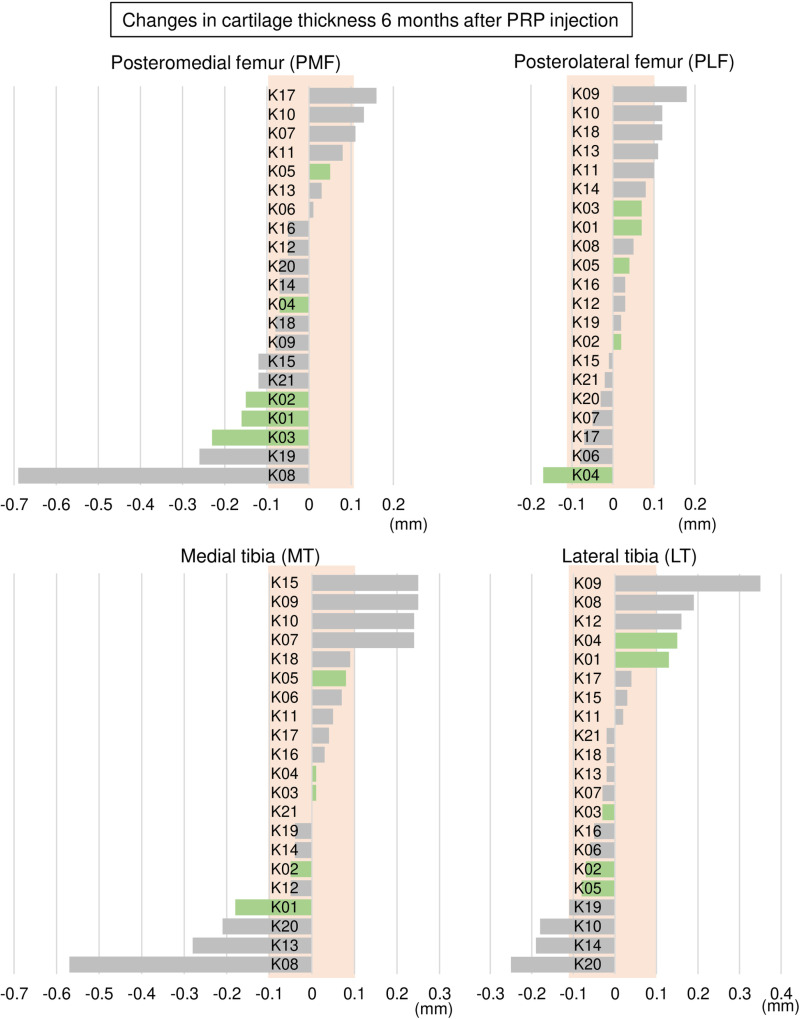
Changes in cartilage thickness 6 months after PRP injection in the tibiofemoral joint. For each region, the changes are shown in decreasing order, beginning with the greatest change. Positive values indicate thickening, while negative values indicate thinning. Green bars represent KL 0 and 1, while gray bars represent KL 3 and 4. The orange background indicates the range in which the absolute value of change is within 0.1 mm.

**Fig 6:**
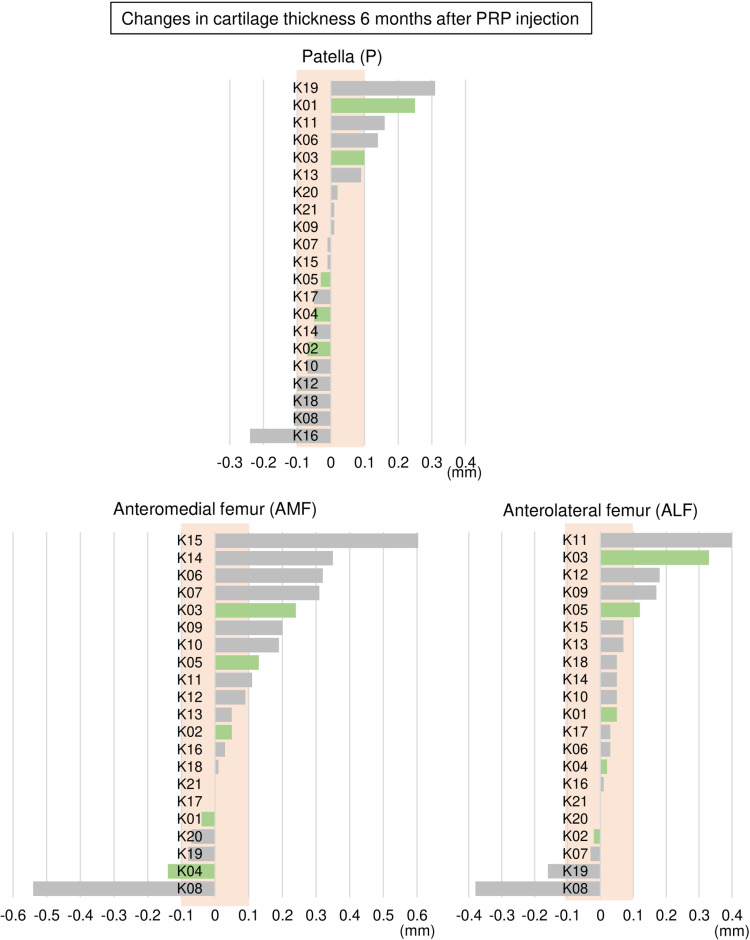
Changes in cartilage thickness 6 months after PRP injection in the patellofemoral joint. For each region, the changes are shown in decreasing order, beginning with the greatest change. Positive values indicate thickening, while negative values indicate thinning. Green bars represent KL 0 and 1, while gray bars represent KL 3 and 4. The orange background indicates the range in which the absolute value of change is within 0.1 mm.

Figs 3 and 4 shows reconstructed 3D MRI images of the knee cartilage and ROIs for all subjects. In the PMF region, the change in cartilage thickness over 6 months (ΔThickness) increased in 7 knees (33%) and decreased in 14 knees (67%) (Figs 5 and 6). Considering the variations with an absolute magnitude greater than 0.1 mm as changes exceeding the margin of error, ΔThickness > 0.1 mm (which is considered to indicate an increase beyond the detection threshold) was observed in 3 knees (14%), -0.1 mm ≤ ΔThickness ≤ 0.1 mm (which is considered to be within the margin of error) in 11 knees (52%), and ΔThickness <-0.1 mm (which is considered to indicate a decrease beyond the detection threshold) in 7 knees (33%).

**Fig 7:**
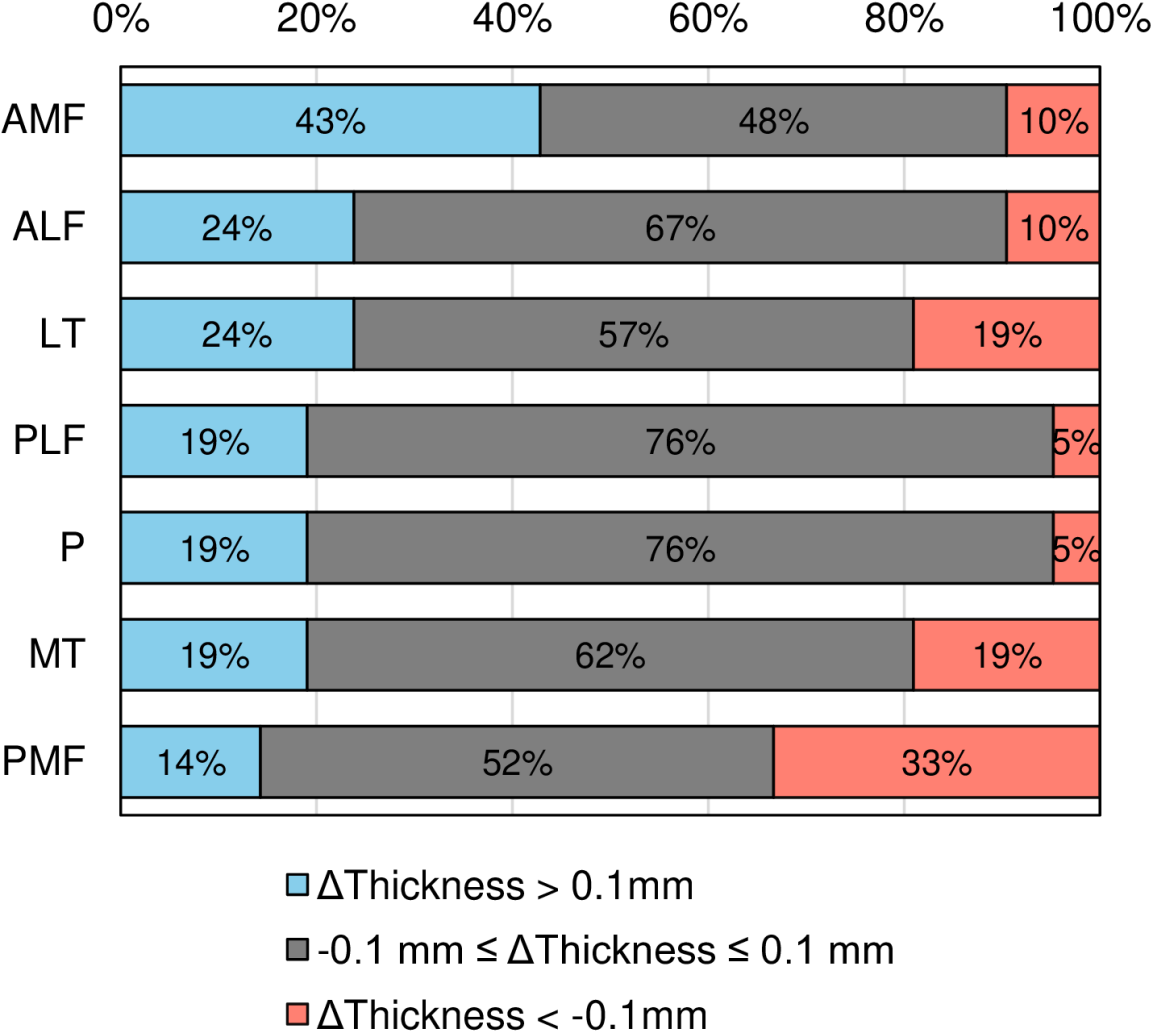
Proportions of cartilage thickness changes by threshold. Changes in cartilage thickness 6 months after PRP injections were categorized using three thresholds. The change in cartilage thickness over 6 months is denoted as ΔThickness. Considering the variations with an absolute magnitude greater than 0.1 mm as changes exceeding the margin of error, ΔThickness > 0.1 mm is considered to indicate an increase beyond the detection threshold, -0.1 mm ≤ ΔThickness ≤ 0.1 mm is considered to be within the margin of error, and ΔThickness <-0.1 mm is considered to indicate a decrease beyond the detection threshold.

The proportions of ΔThickness > 0.1 mm for all seven regions are summarized in Fig 7. The highest proportion was observed in the anterior medial femoral (AMF) region at 43%, followed by the anterior lateral femoral (ALF) and lateral tibial (LT) regions at 24%, then the posterior lateral femoral (PLF), patellar (P), and medial tibial (MT) regions at 19%, with the posterior medial femoral (PMF) region showing the lowest proportion at 14%. Conversely, ΔThickness <-0.1 mm was observed in the PMF region at 33%, followed by the MT and LT regions at 19%, and then the AMF and ALF regions at 10%, with the PLF and P regions showing the lowest proportion at 5%.

### Relationships of cartilage thickness changes with age and KL grade

Scatter plots did not show any apparent relationship between patient age and changes in cartilage thickness across seven regions (S1 Fig). Correlation analysis between age and changes in cartilage thickness, adjusted for multiple testing using Bonferroni correction (p=0.00714, derived from 0.05/7), found no significant correlations, as all p-values exceeded this threshold (S1 Table). Similarly, correlation analysis between the KL grade and changes in cartilage thickness showed no significant correlations following the same adjustment method (S2 Fig, S2 Table).

### Changes in KOOS 6 months after PRP injection

**Fig 8:**
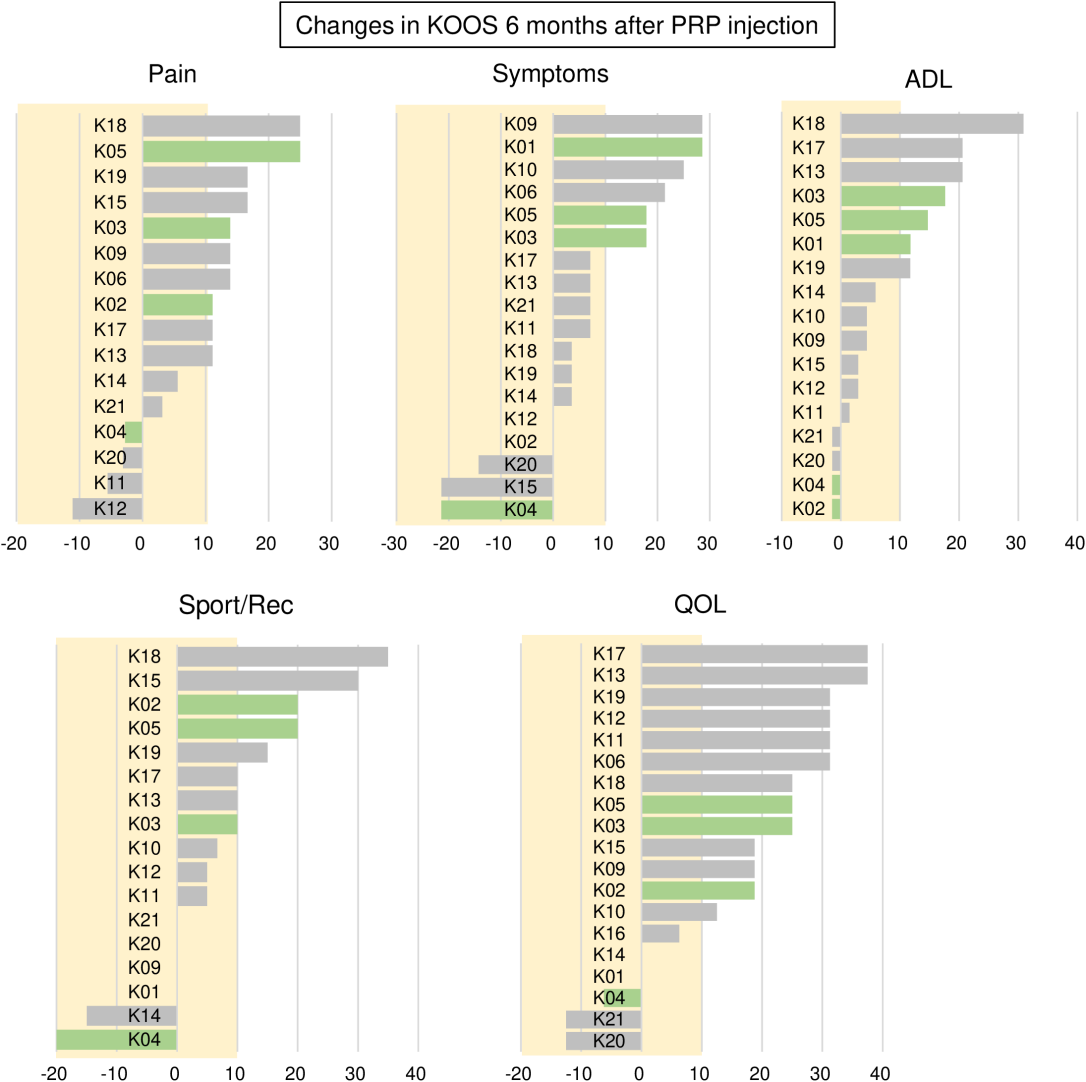
Changes in KOOS 6 months after PRP injection. For each subscale, the changes are shown in order, beginning with the greatest change. Positive values indicate improvement, while negative values indicate deterioration. Green bars represent KL 0 and 1, while gray bars represent KL 3 and 4. The yellow background indicates the range in which the value of change is below 10.

In the pain subscale, scores increased in 12 knees and decreased in 4 knees (Fig 8). Considering a minimal clinically important difference (MCID) of 10, 10 knees (63%) showed changes equal to or exceeding this threshold. The proportions of knees showing changes greater than or equal to 10 in other subscales were: 32% for symptoms, 41% for function in activities of daily living (ADL), 47% for sport and recreation function (Sport/Rec), and 68% for quality of life (QOL).

### No correlation between changes in cartilage thickness and KOOS values

**Fig 9:**
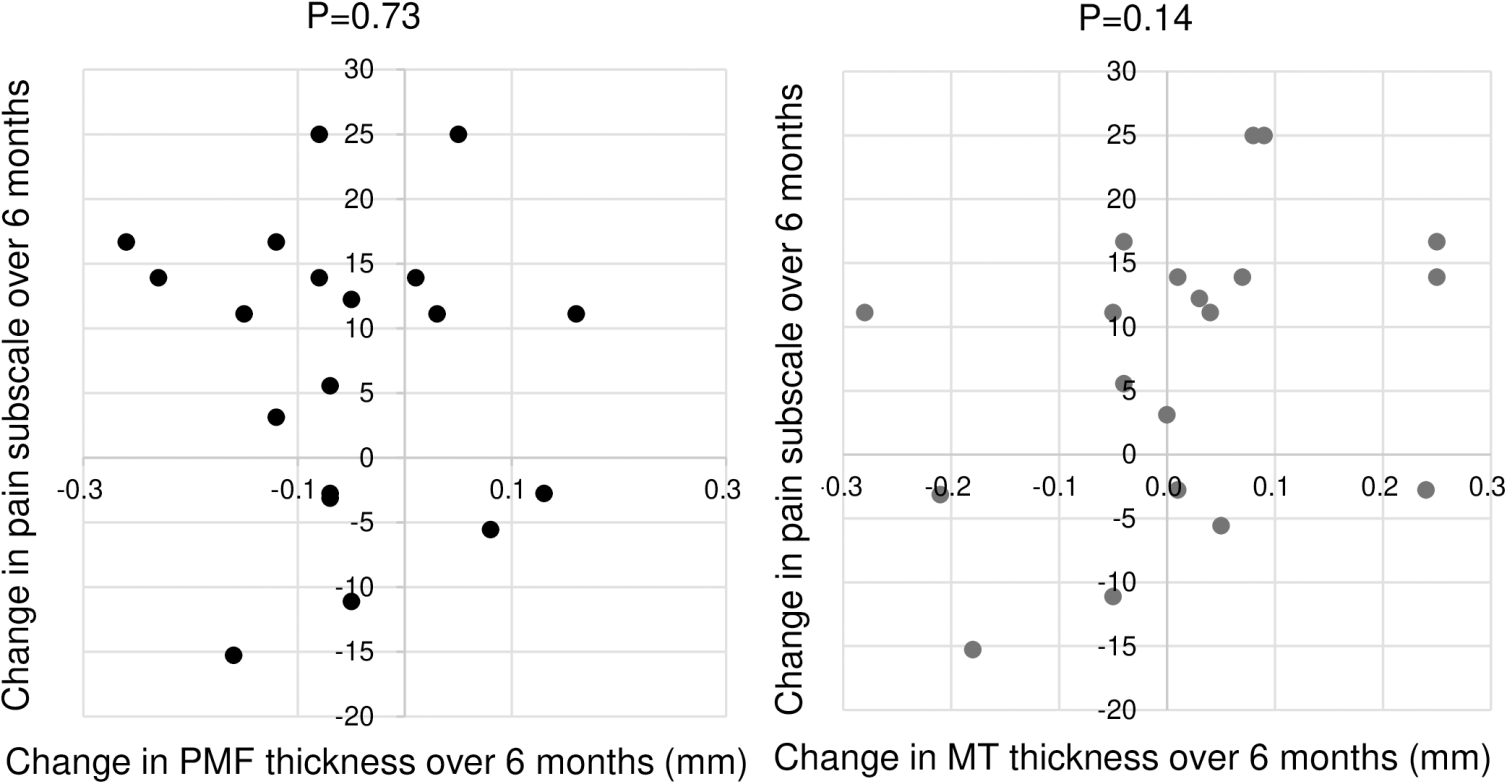
Scatter plots showing the relationship between the change in cartilage thickness and changes in KOOS pain scores over 6 months. Representative plots for the PMF and MT regions are shown. No correlation was found between the change in cartilage thickness and the change in KOOS pain score in either the PMF or MT regions.

No significant correlation was found between the change in PMF thickness over 6 months and changes in the pain subscale over 6 months (Fig 9). Similarly, no significant correlation was observed between the change in MT thickness over 6 months and changes in the pain subscale over 6 months. Correlation analyses conducted for 35 combinations of cartilage thickness changes in seven regions and five KOOS subscales did not reveal significant correlations in any of these combinations. Detailed p-values for all correlation analyses showed some comparisons with relatively low p-values, such as between PLF thickness and symptoms (p=0.029) and between P thickness and symptoms (p=0.054); however, none reached statistical significance after Bonferroni correction for multiple testing (adjusted significance level: p=0.00143) (S3 Table).

## Discussion

We evaluated in detail the changes in cartilage thickness in knees with medial knee osteoarthritis six months after PRP injection using our recently developed 3D-MRI evaluation system [18]. The proportion of cartilage thickness increase (>0.1 mm) was highest in the AMF region (43%), followed by the ALF and LT regions (24%); and then the PLF, P, and MT regions (19%), and was lowest in the PMF region (14%). These findings indicate a regional variability in the response to PRP injection. Importantly, the PMF and MT regions, which are often significantly affected in medial knee osteoarthritis, showed the least improvement in cartilage thickness. This suggests that the effect of PRP on increasing cartilage thickness in medial knee osteoarthritis is limited.

Previous 2D-MRI studies on the effects of PRP on cartilage structure in knee osteoarthritis have yielded mixed results. For example, Kon et al. found that PRP (APS) prevented the worsening of bone marrow edema and osteophytes, compared to saline treatment, after one year [7], while Lisi et al. reported significant cartilage improvement in their PRP group, compared to controls, after 6 months [8]. However, Buendía-López et al. observed no difference in cartilage thickness progression in patients treated with NSAIDs, hyaluronic acid, or PRP after one year [6]. Similarly, Bansal et al., in a comparison of 64 patients treated with PRP and 68 patients treated with hyaluronic acid (HA), found no increase in cartilage thickness in either group [5]. These contradictory findings, which were based on 2D-MRI analyses, have led to a current lack of consensus regarding the cartilage structure–improving effects of PRP in knee osteoarthritis.

Unfortunately, 3D-MRI evaluations of PRP effects on cartilage structure in knee osteoarthritis are limited. Raeissadat et al. conducted a double-blind study on 46 patients with bilateral knee osteoarthritis following the injection of PRP in one knee and saline in the other every 4 weeks [11]. After 8 months, they reported a significant increase in patellofemoral joint cartilage volume, from 1,041 μL to 1,337 μL, in PRP-treated knees. However, the accuracy and objectivity of this quantitative assessment are questionable, as the volume was calculated by obtaining the cartilage area through manual segmentation and multiplying that area value by the slice thickness. Bennell et al. compared 144 patients with osteoarthritis who received PRP injections with 144 who received saline injections [10,11]. After one year, they found no significant difference in the mean change (-1.4% for PRP and -1.2% for saline) in the medial tibial cartilage volume. Their methodology involved having a single rater use OsiriX software to manually draw disarticulation contours around the cartilage boundaries observed on sagittal MRI images, raising concerns about objectivity. The limitations of these studies highlight the need for more robust, objective 3D-MRI evaluation methods designed to provide accurate assessments of PRP effects on cartilage structure in knee osteoarthritis.

We have demonstrated the precision of cartilage thickness measurements using 3D-MRI analysis systems many times in multiple reports. For example, in our study by Sekiya et al., we had ten healthy volunteers undergo MRI twice in the same day [18]. That study clarified that the interscan measurement error of knee cartilage thickness was less than 0.10 mm in all 9 regions, including the AMF, PMF, ALF, PLF, MT, and LT. Similarly, our study by Katano et al. evaluated the variations in knee cartilage thickness measurements in ten healthy volunteers using automated methods and MRI instruments from five vendors [17]. The volunteers underwent MRI scans 5 times using equipment from five different companies, and no exceptional trends attributable to a specific instrument model were observed. Of the 350 measurements (= 10 subjects x 7 regions x 5 vendors), the inter-measurement error was ≤0.05 mm in 53%, ≤0.10 mm in 75%, and ≤0.20 mm in 95%. In our subsequent study by Katano et al., we analyzed the interscan measurement error in 50 knee osteoarthritis patients (25 KL1–2, 25 KL3–4) who were all scanned twice using five different MRI models. In the KL1–4 group, of the 175 measurements (= 5 subjects x 7 regions x 5 vendors), 37%, 67%, and 93% had interscan errors ≤ 0.05 mm, ≤ 0.10 mm, and ≤ 0.20 mm, respectively (in submission). Based on the high precision indicated by these findings, we set the error range for the current study at 0.1mm.

The previous PRP preparation methods and administration protocols for knee osteoarthritis treatment lacked standardization across studies [24], thereby creating a significant challenge in evaluating treatment efficacy. Various factors, including platelet concentration, leukocyte content, activation methods, and administration frequency, can potentially influence therapeutic outcomes [25]. In our study, we used the nStride Autologous Protein Solution (APS) system (Zimmer Biomet) and followed the manufacturer’s standardized preparation protocol (as detailed in the Methods section). While our findings provide insights into cartilage thickness changes induced following this specific protocol, the results may not be generalizable to other PRP preparation methods. Future studies would benefit from systematic evaluation of these protocol variables using standardized preparation methods and detailed reporting of cellular composition to establish optimal treatment protocols and facilitate meaningful comparisons across studies.

We found no correlation between changes in cartilage thickness and KOOS scores in the present study. This lack of correlation adds to the complex and often discordant relationship between cartilage structural changes and clinical symptoms in osteoarthritis, as evidenced in both animal [26] and human studies [27]. The available literature contains inconsistent results regarding this relationship in patients with knee osteoarthritis who receive PRP injections. For instance, Raeissadat et al. observed significant improvements in both the Western Ontario and McMaster Universities Osteoarthritis Index (WOMAC) and visual analog scale (VAS) scores, along with increased patellofemoral cartilage volume determined by 3D MRI assessments [11]. Similarly, Baki et al. reported improvements in WOMAC scores, with significant increases in cartilage thickness determined using ultrasound measurements, after PRP injection [28]. These contradictory findings suggest that the relationship between structural changes and clinical outcomes in PRP treatment remains unclear and may be influenced by multiple factors, including measurement methods and patient characteristics.

The mechanism underlying region-specific changes in cartilage thickness also remains unclear, although various growth factors and cytokines contained in PRP could theoretically promote cartilage regeneration [29]. The observed regional variations might be influenced by many factors, including local mechanical stress distributions, varying concentrations of growth factors, or differences in tissue responsiveness across the joint. Furthermore, increased cartilage thickness in specific regions might not necessarily translate into improved joint function, as the cartilage changes could potentially alter joint biomechanics. These diverse findings suggest that, while PRP may affect both clinical symptoms and cartilage structure, the relationship between these changes is complex and requires careful interpretation in the context of overall joint biomechanics. Further research is needed to elucidate the long-term effects of PRP and the mechanisms underlying its therapeutic effects on cartilage.

The quality of the regenerated cartilage tissue is another crucial consideration when evaluating the therapeutic outcomes of osteoarthritis treatments. While our study quantitatively assessed cartilage thickness changes, effective cartilage regeneration therapies should aim to restore more than just the cartilage volume and include restoration of the biomechanical and structural properties that characterize healthy native cartilage. Advanced imaging techniques, such as T2 mapping, T1rho, and dGEMRIC sequences, could provide valuable insights into the compositional and structural properties of the cartilage tissue that forms following PRP treatment. These methods can assess important qualitative parameters, including collagen fiber organization, proteoglycan content, and water distribution within the cartilage matrix [30]. Future studies combining both quantitative measurements of cartilage thickness and qualitative assessment of tissue properties would offer a more comprehensive evaluation of cartilage regeneration outcomes after PRP intervention.

The changes in cartilage thickness observed in the present study following PRP treatment did not reach statistical significance, which warrants careful consideration of several important factors. One is that the biological response to PRP appears to be heterogeneous, with some patients showing increased cartilage thickness and others showing decreased thickness over the 6 month observation period. This variability may be attributable to the complex pathophysiology of osteoarthritis, as patients present with different stages of disease progression and varying degrees of cartilage degradation. Furthermore, because the observed changes in cartilage thickness were relatively small and approached the inherent measurement error of our MRI-based technique, this made statistical validation particularly challenging. These observations suggest that future studies would benefit from larger sample sizes, longer follow-up periods, and potential stratification of patients based on disease characteristics for a better elucidation of the effects of PRP on cartilage thickness.

The heterogeneity of our study population represents a notable limitation of this research. Our participants exhibited considerable variation in both age and disease severity, which is common in osteoarthritis populations, but may have influenced our ability to detect treatment effects. Additionally, while age-related variations in cartilage metabolism could potentially influence treatment outcomes [31], our small sample size of only 21 cases precluded any meaningful subgroup analyses by age. Given that osteoarthritis is a highly diverse disease with varying manifestations and progression patterns, a more homogeneous study population might have provided clearer insights into PRP effectiveness.

We acknowledge two additional limitations of this study. First, both knees were analyzed in six patients. Ideally, all analyses should have been conducted on single knees, but we prioritized increasing the sample size. Second, in setting the ROIs, we had to copy from the same knee scanned twice, or we had to manually modify them, in some cases. We noticed that our system sometimes fails to set appropriate ROIs in cases with cartilage defects. This issue is expected to improve through updates using AI learning. Future studies would benefit from larger sample sizes with more stringent inclusion criteria to create more uniform study groups and potentially stratifying analyses by age groups and disease severity. That type of approach would enhance the statistical power and provide more precise estimates of PRP effects within specific patient subgroups, ultimately leading to better targeted therapeutic applications.

In conclusion, our findings reveal a significant limitation in the regenerative effect of PRP on cartilage thickness, particularly in regions most affected by medial osteoarthritis. The posteromedial femoral and medial tibial areas showed minimal improvement, with less than 20% of the cases experiencing increased cartilage thickness. While PRP injection may positively affect certain knee regions, its effects on the areas most impacted by medial osteoarthritis appear limited. This suggests that the therapeutic potential of PRP for cartilage regeneration in medial knee osteoarthritis may require further investigation to obtain a more complete understanding of its actual efficacy and optimal application.

## Supporting information

S1 Fig Scatter plots showing the relationships between age and changes in cartilage thickness over 6 months for seven regions. (TIF)

S2 Fig Scatter plots showing the relationships between KL grade and changes in cartilage thickness over 6 months for seven regions. (TIF)

S1 Table P-value of correlation analysis between age and changes in cartilage thickness for seven regions. (DOCX)

S2 Table P-value of correlation analysis between KL grade and changes in cartilage thickness for each region. (DOCX)

S3 Table P-value of correlation analysis between changes in cartilage thickness for each region and changes in each KOOS subscale. (DOCX)

## Abbreviations

APS: autologous protein solution
DICOM: digital imaging and communications in medicine
MRI: magnetic resonance imaging
3D MRI: three-dimensional MRI
2D MRI: two-dimensional MRI
OA: osteoarthritis
PDW: proton-density weighted
PRP: platelet-rich plasma
SPGR: spoiled gradient
