# Supplementary figures and images for: 3D-MRI analysis of cartilage thickness changes after PRP injection in medial knee osteoarthritis: A preliminary report
Source: PLoS One. 2025 Apr 30;20(4):e0321067. doi: 10.1371/journal.pone.0321067 (PMC12043159; doi:10.1371/journal.pone.0321067)

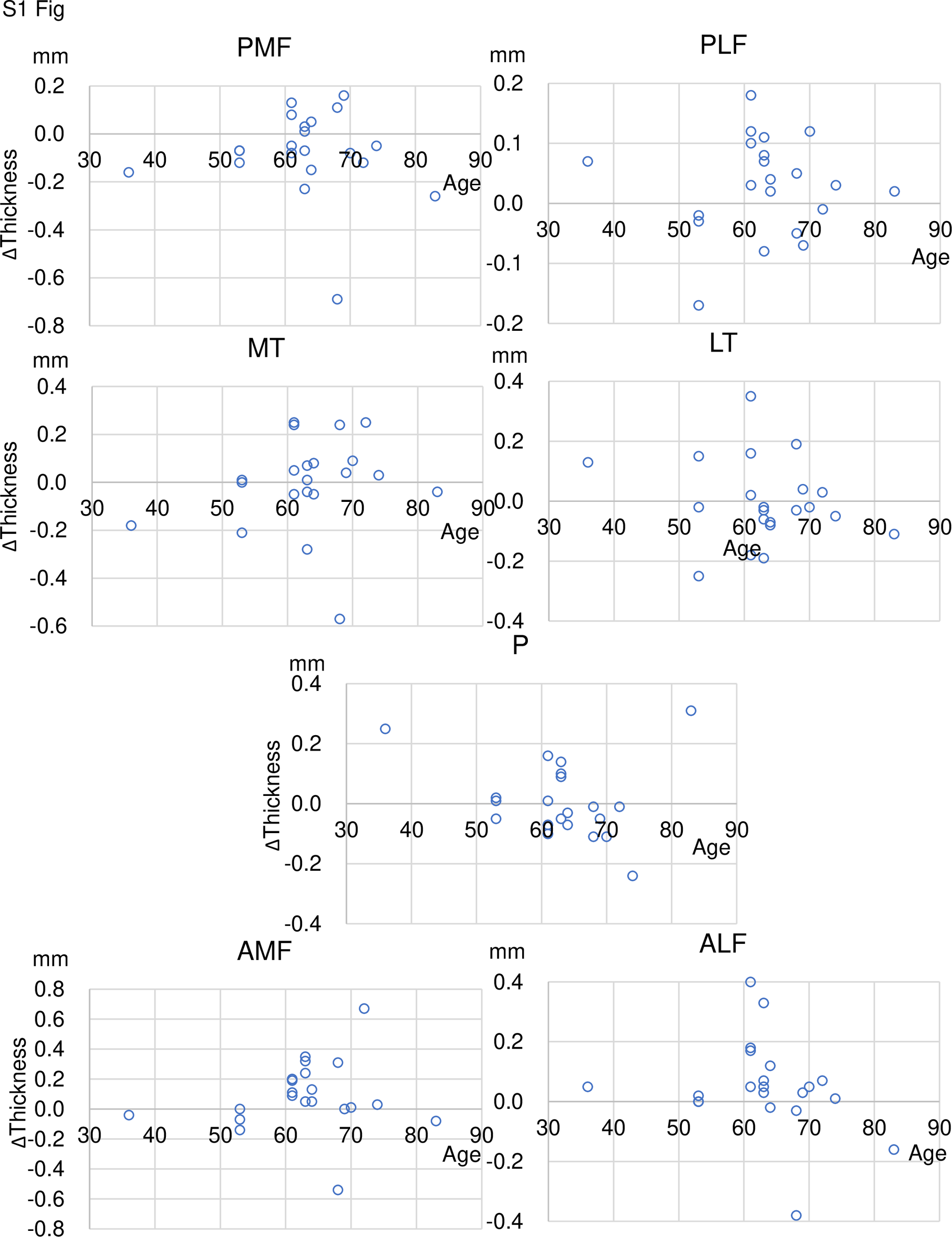

Supplement: S1 Fig — (TIF) [file pone.0321067.s001.tif]

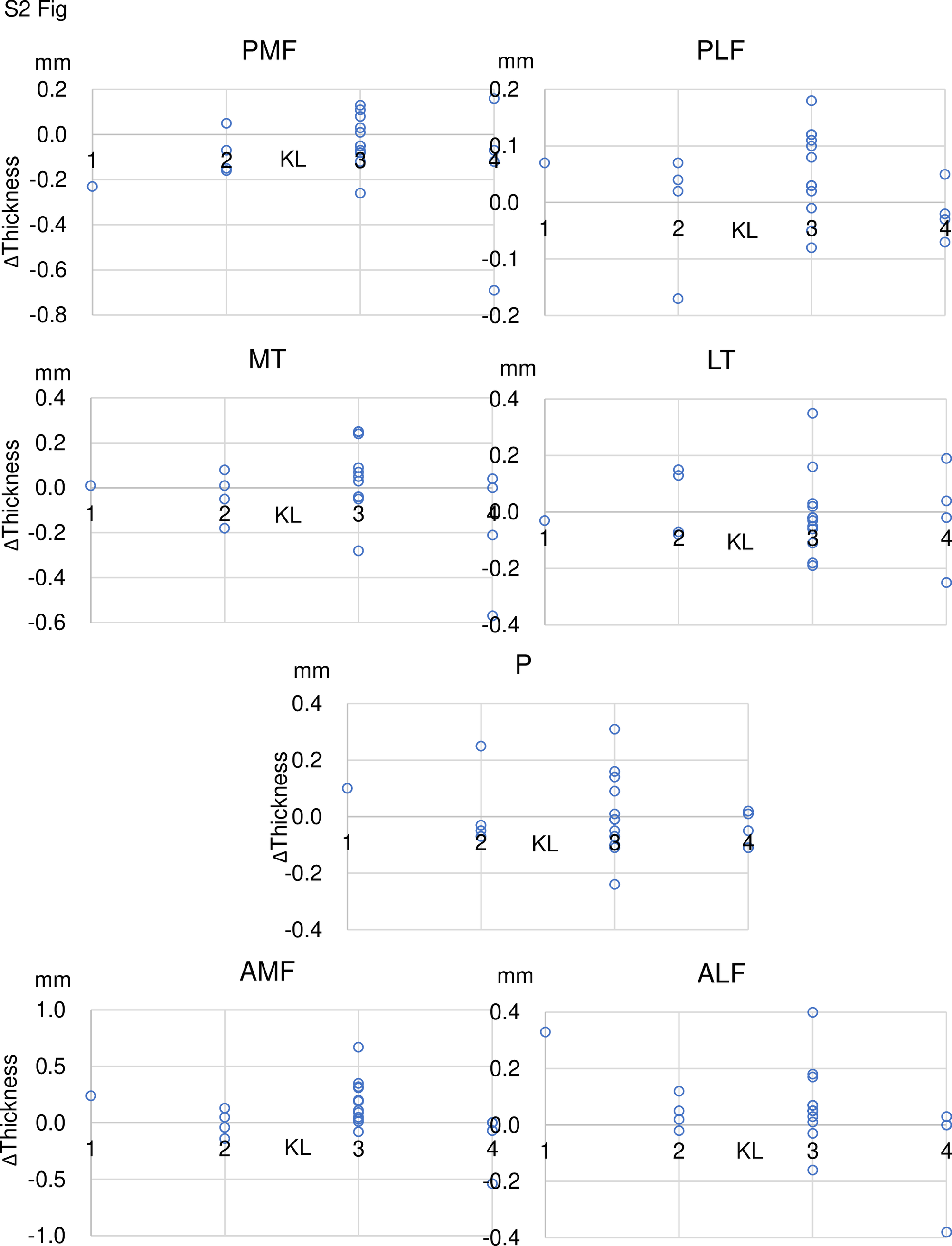

Supplement: S2 Fig — (TIF) [file pone.0321067.s002.tif]
