# Supplementary material for: 3D-MRI analysis of cartilage thickness changes after PRP injection in medial knee osteoarthritis: A preliminary report
Source: PLoS One. 2025 Apr 30;20(4):e0321067. doi: 10.1371/journal.pone.0321067 (PMC12043159; doi:10.1371/journal.pone.0321067)
Supplement: S1 Table — (DOCX) [file pone.0321067.s003.docx]

| **S1 Table.** P-value of correlation analysis between age and changes in cartilage thickness for seven regions. | | | | | | | |
| --- | --- | --- | --- | --- | --- | --- | --- |
|  | PMF | PLF | MT | LT | P | AMF | ALF |
| Age | 0.457 | 0.609 | 0.281 | 0.416 | 0.588 | 0.878 | 0.338 |
| After adjusting the significance level to p=0.00714 (=0.05/7) using Bonferroni correction (due to multiple testing of 7 items), no significant correlations were found as all p-values exceeded this threshold. | | | | | | | |
