# Supplementary material for: 3D-MRI analysis of cartilage thickness changes after PRP injection in medial knee osteoarthritis: A preliminary report
Source: PLoS One. 2025 Apr 30;20(4):e0321067. doi: 10.1371/journal.pone.0321067 (PMC12043159; doi:10.1371/journal.pone.0321067)
Supplement: S2 Table — (DOCX) [file pone.0321067.s004.docx]

| **S2 Table.** P-value of correlation analysis between KL grade and changes in cartilage thickness for each region. | | | | | | | |
| --- | --- | --- | --- | --- | --- | --- | --- |
|  | PMF | PLF | MT | LT | P | AMF | ALF |
| KL | 0.049 | 0.361 | 0.168 | 0.513 | 0.755 | 0.629 | 0.040 |
| After adjusting the significance level to p=0.00714 (=0.05/7) using Bonferroni correction (due to multiple testing of 7 items), no significant correlations were found as all p-values exceeded this threshold. | | | | | | | |
