# Supplementary material for: 3D-MRI analysis of cartilage thickness changes after PRP injection in medial knee osteoarthritis: A preliminary report
Source: PLoS One. 2025 Apr 30;20(4):e0321067. doi: 10.1371/journal.pone.0321067 (PMC12043159; doi:10.1371/journal.pone.0321067)
Supplement: S3 Table — (DOCX) [file pone.0321067.s005.docx]

| **S3 Table.** P-value of correlation analysis between changes in cartilage thickness for each region and changes in each KOOS subscale. | | | | | | | |
| --- | --- | --- | --- | --- | --- | --- | --- |
|  | PMF | PLF | MT | LT | P | AMF | ALF |
| Symptoms | 0.568 | 0.029 | 0.626 | 0.491 | 0.054 | 0.817 | 0.261 |
| Pain | 0.734 | 0.675 | 0.141 | 0.564 | 0.535 | 0.242 | 0.488 |
| ADL | 0.705 | 0.215 | 0.731 | 0.981 | 0.564 | 0.806 | 0.846 |
| Sport/Rec | 0.854 | 0.332 | 0.275 | 0.553 | 0.456 | 0.348 | 0.766 |
| QOL | 0.262 | 0.304 | 0.555 | 0.496 | 0.432 | 0.409 | 0.231 |
| After adjusting the significance level to p=0.00143(=0.05/35) using Bonferroni correction (due to multiple testing of 35 items), no significant correlations were found as all p-values exceeded this threshold. | | | | | | | |
